# Swine acute diarrhea syndrome coronavirus-related viruses from bats show potential interspecies infection

**DOI:** 10.1128/jvi.02240-24

**Published:** 2025-11-19

**Authors:** Yun Luo, Qian-Chun Gong, Yu-Lin Yao, Ying Chen, Hao-Rui Si, Xing Xie, Yong-Le Yang, Zhi-Xin Feng, Ren-Di Jiang, Yao-Wei Huang, Xin-Hua Lin, Peng Zhou

**Affiliations:** 1 The Fifth Affiliated Hospital of Guangzhou Medical University, Guangzhou Laboratory Clinical Base, Guangzhou Medical University, Guangzhou, China; 2 State Key Laboratory of Genetic Engineering, Greater Bay Area Institute of Precision Medicine (Guangzhou), School of Life Sciences, Zhongshan Hospital, Fudan University 92323, Shanghai, China; 3 Hubei Key Laboratory of Cognitive and Affective Disorders, Wuhan Institute of Biomedical Sciences, School of Medicine, Jianghan University 470004, Wuhan, China; 4 Changping Laboratory 662243 , Beijing, China; 5 Guangzhou Medical University 26468, Guangzhou, China; 6 Key Laboratory for Veterinary Bio-Product Engineering, Ministry of Agriculture and Rural Affairs, Institute of Veterinary Medicine, Jiangsu Academy of Agricultural Sciences 668638 , Nanjing, China; 7 Xianghu Laboratory 665999 , Hangzhou, China; 8 State Key Laboratory for Animal Disease Control and Prevention, South China Agricultural University 12526, Guangzhou, China; Emory University School of Medicine, Atlanta, Georgia, USA

**Keywords:** swine acute diarrhea syndrome coronavirus, SADS-related coronavirus, spike, risk assessment, pathogenicity, cross-neutralization

## Abstract

Swine acute diarrhea syndrome coronavirus (SADS-CoV) is a bat-originated virus causing severe diseases in piglets. Since the 2016 outbreak, diverse SADS-related CoVs (SADSr-CoVs) have been detected in *Rhinolophus* bats in China and Southeast Asia, but their potential interspecies infection and pathogenicity remain unknown. Herein, we sequenced the spike (S) genes of bat SADSr-CoVs and classified them into four genotypes. We constructed an infectious SADS-CoV cDNA clone (rSADS-CoV) and nine recombinant viruses by replacing the SADS-CoV S gene with that of bat SADSr-CoVs. Recombinant SADSr-CoVs could replicate efficiently in respiratory and intestinal cell lines and human- and swine-derived organoids and caused varying tissue damage and mortality in suckling mice. These viruses can be classified into at least five serotypes based on cross-neutralization assays. Our findings highlight the potential risk of interspecies infection and provide important information for future surveillance of these bat viruses.

**IMPORTANCE:**

Over the last 20 years, several bat-originated coronaviruses (CoVs), including SARS-CoV, MERS-CoV, and , have caused millions of deaths and severely disrupted global health systems, highlighting the need to investigate bat CoV spillover risks. SADS-CoV, another bat-derived CoV highly pathogenic to piglets, threatens the swine industry and exhibits broad cell tropism, underscoring the need to study these highly diverse viruses with potential for interspecies infection and pathogenicity. As part of our effort to understand these viruses, we developed a framework to characterize them in cell lines and organoids derived from swine and humans, as well as in suckling mice. Additionally, we performed serum cross-neutralization to classify bat SADSr-CoV serotypes, which could guide the development of broad-spectrum vaccines against SADSr-CoVs.

## INTRODUCTION

Coronaviruses (CoVs) are enveloped, positive-sense, single-stranded RNA viruses that cause mild-to-severe respiratory diseases in humans and gastrointestinal diseases in animals. Bats carry the highest proportion of CoVs and ancestor strains of some human CoVs, including the severe acute respiratory syndrome coronavirus (SARS-CoV), Middle East respiratory syndrome coronavirus (MERS-CoV), and severe acute respiratory syndrome coronavirus 2 (SARS-CoV-2) (1–6). Domestic or game animals play an important role in virus transmission from bats to humans, e.g., SARS-CoV through civets in the wet market and MERS-CoV through dromedaries (7, 8).

CoV transmission from bats to other animals has been well documented (9–12). For example, bat-origin swine acute diarrhea syndrome coronavirus (SADS-CoV) caused a large-scale outbreak of fatal diarrhea in suckling piglets in 2016 in Guangdong Province, China (13–15). Since the outbreak of SADS-CoV in 2016, this virus has been detected in multiple pig farms in five provinces in southern and central China, including Guangdong, Fujian, Jiangxi, Guangxi, and Henan. The recent reemergence of SADS-CoV infection in pig herds has led to small- and medium-scale outbreaks of swine diarrhea (16–20). Despite the absence of an ongoing regional epidemic, the virus persists and continues to spread discreetly (21).

Retrospective surveillance suggested that SADS-CoV was a recent spillover event from bats, as closely related CoVs were detected in bat samples collected in 2016 from the same region as the SADS outbreak (15). Furthermore, a large number of genetic diversity of swine acute diarrhea syndrome-related coronaviruses (SADSr-CoVs) or HKU2-related CoV were detected in *Rhinolophus* bats, including *R. affinis*, *R. sinicus*, *R. rex*, and *R. pusillus* across several provinces in southern China (22–24). These raise the question of whether diverse bat SADSr-CoV could cause spillover. Previous studies have shown that SADS-CoV has wide tissue tropism in different hosts, including primary human lung and intestinal cells. They can also cause morbidity and mortality in suckling pigs and mice, posing a significant risk of zoonotic spillover in humans (25, 26). However, little is known about the pathogenicity of bat SADSr-CoV.

The CoV spike (S) protein is essential for entry into host cells; hence, it is a determinant of tissue tropism. The S protein generally consists of two functional subunits, S1 and S2, which mediate receptor engagement and membrane fusion, respectively. The S1 subunit can be further subdivided into two subdomains, the N-terminal domain (S1-NTD) and the C-terminal domain (S1-CTD), which may bind carbohydrates or transmembrane proteins as viral receptors (27). In a previous study, we found genetically diverse bat SADS-CoVs with highly diverse S proteins (15).

In this study, we analyzed bat SADS-CoV S genes from archived samples and constructed an infectious SADS-CoV cDNA clone and various recombinant viruses by replacing the SADS-CoV S gene with that of bat SADSr-CoV. We also developed a framework to fully characterize these viruses in cell lines and organoids derived from swine and humans, as well as suckling mice. Our results indicate that viruses expressing the bat SADSr-CoV S protein have potential spillover to animals and humans, as evidenced by their efficient replication in the tested cell lines and organoids and their pathogenicity in the tested animals.

## RESULTS

### Variations in bat SADSr-CoV spike proteins

**Fig 1:**
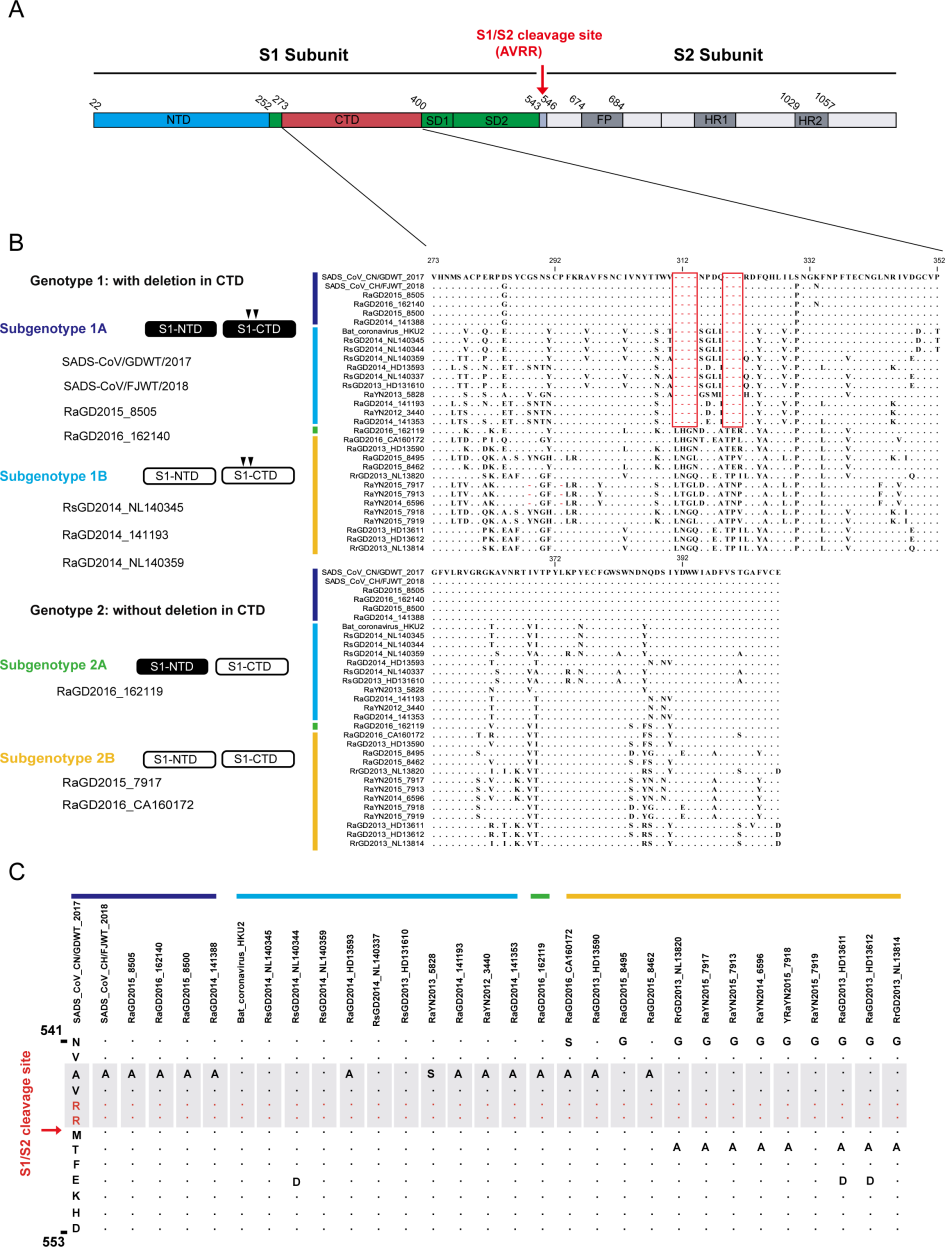
Sequence comparison of spike proteins between bat SADSr-CoV and SADS-CoV. (**A**) Schematic of SADS-CoV S protein sequence. FP, fusion peptide; HR, heptad repeat; S1-CTD, C-terminal domain of S1; S1-NTD, N-terminal domain of S1; SD1, subdomain 1; SD2, subdomain 2. (**B**) Representative subgenotypes of S1 from SADS-CoV and bat SADSr-CoVs. The left panel indicates the coding regions of the S1-NTD and S1-CTD. Bat SADSr-CoVs that share high similarity (>95%) with SADS-CoV spike are indicated by black boxes, and those that share less sequence similarity to SADS-CoV are indicated by hollow boxes. The deletions in the S1-CTD are indicated by two inverted triangles. These viruses are initially classified into two genotypes based on the absence or presence of deletions in S1-CTD. Further subdivisions into two genotypes are based on variations in the S1-NTD and S1-CTD amino acid sequences of genotypes 1 and 2, respectively. The right panel indicates the alignment of SADS-CoV S1-CTD (amino acids 273–400) with homologous regions of bat SADSr-CoVs using ClustalW. The deleted amino acid residues in the S1-CTD are marked with red boxes. SADS-CoV/GDWT/2017 and SADS-CoV/FJWT/2018 were identified from diarrhea piglets collected in Guangdong in 2017 and Fujian Province in 2018, respectively. Bat SADSr-CoV identified in this study was named based on the bat species, followed by the sampling location, collection year, and sample ID. (**C**) Furin-cleavage site alignment between bat SADSr-CoV and SADS-CoV. The arginine sites are shown in red, and the similar furin-cleavage sites are gray shaded.

We sequenced 69 bat SADSr-CoV S genes from bat samples collected from 2012 to 2015 in China and compared them with those of SADS-CoV and previously reported bat SADSr-CoV. These S proteins shared a high amino acid identity in the S2 subunit and contained similar furin-cleavage sites while displaying high diversity in their S1 subunits (Fig. 1; Fig. S1). They were categorized into two distinct genotypes based on the presence or absence of deletions in S1-CTD: genotype 1, including SADS-CoV, which has two deletions in the S1-CTD (311–314 aa and 319–321 aa), and genotype 2, which has no deletions in S1-CTD (Fig. 1). They were further divided into four subgenotypes (1A/B and 2A/B) based on variations in their S1-NTD and S1-CTD amino acid sequences. Subgenotype 1A strains, including SADS-CoV and two representative bat SADSr-CoVs (sample IDs 8505 and 162140), are highly similar to each other in both S1-NTD and S1-CTD regions, with 8505-CoV being the most similar to SADS-CoV (98.9% amino acid sequence identity) among all bat SADSr-CoVs reported to date. Subgenotype 1B (sample IDs NL140345, 141193, and NL140359) showed 86.4%–90.6% amino acid identity and more genetic differences from SADS-CoV in both the S1-NTD and S1-CTD regions (Fig. S2; Table S1). The division of subgenotype 2A and 2B was based on the amino acid similarity of the S1-NTD with SADS-CoV. Subgenotype 2A (sample ID 162119) was highly similar to SADS-CoV in the S1-NTD but not in the S1-CTD region, whereas subgenotype 2B (sample IDs 7917 and CA160172) was different in both the S1-NTD and S1-CTD regions (Fig. 1; Fig. S1).

### Development of an infectious cDNA clone of SADS-CoV and corresponding recombinant viruses carrying bat SADSr-CoV spike genes

**Fig 2:**
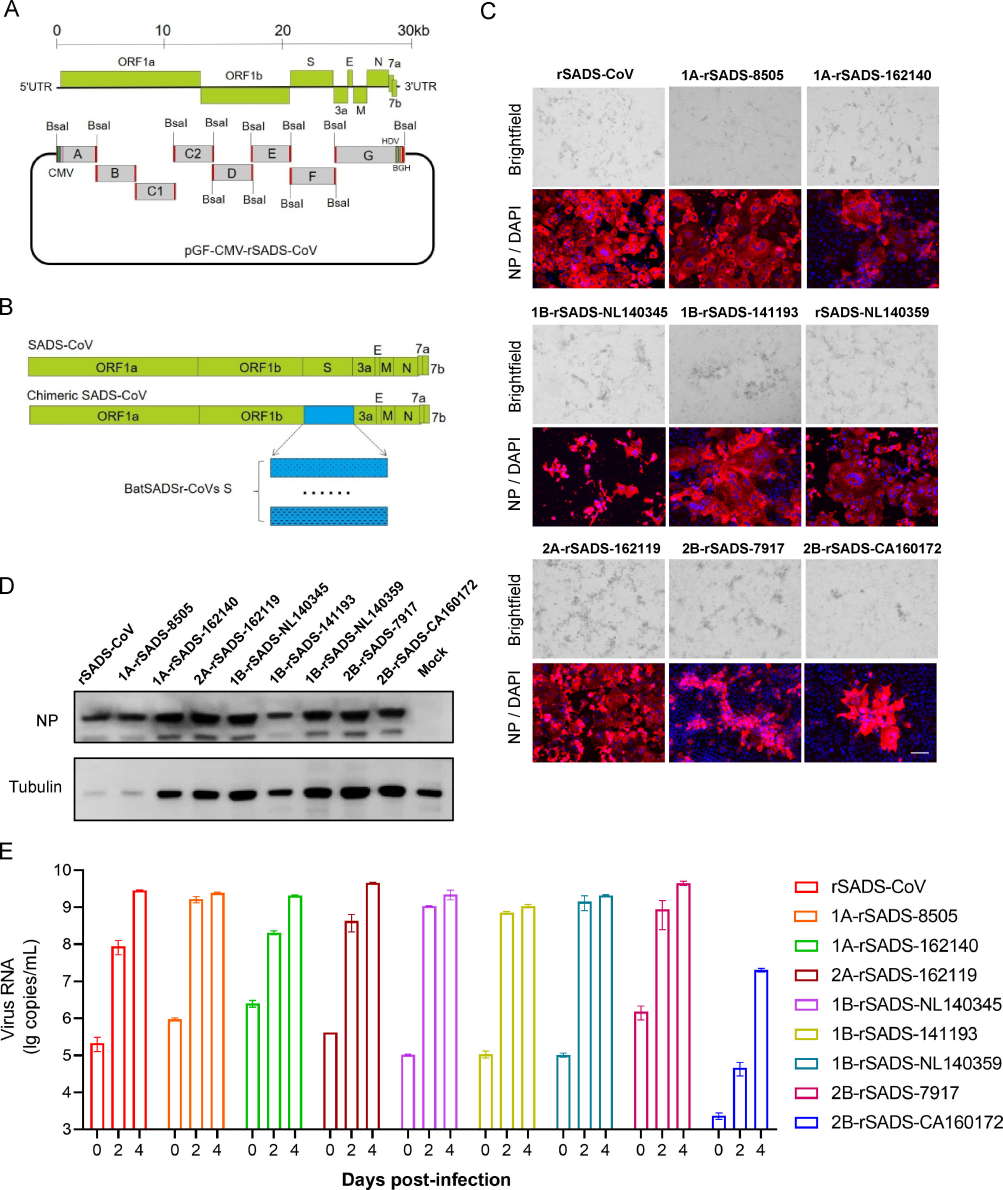
Construction and rescue of SADS-CoV and recombinant SADS-CoVs expressing the spike of bat SADSr-CoVs. (**A**) Schematic representation of constructing SADS-CoV cDNA clone. Eight DNA fragments with overlapping ends were co-transformed into yeast for transformation-associated recombination (TAR) cloning to generate full-length viral cDNA. (**B**) Schematic illustration of constructing the recombinant cDNA clones carrying the bat SADSr-CoV S genes. (**C–E**) Rescue and characterization of recombinant viruses in Huh7. Virus-induced CPE and immunofluorescence staining were conducted at five dpi in Huh7, which was infected with rescued virus passage 2. Cells were stained with rabbit antibody against the SADS-CoV nucleocapsid protein (red) and nuclei were stained with 4′,6-diamidino-2-phenylindole (DAPI) (blue) (**C**), scale bars, 100 µm. The expression of N protein was detected by Western blot at 2 dpi in Huh7 infected with rescued virus passage 3, except 2B-rSADS-CA160172 at 4 dpi (**D**). The supernatant viral RNA was extracted at 0, 2, and 4 dpi from Huh7 infected with rescued virus passage 2 and assessed in duplicate by RT-qPCR (**E**).

The full-length genomic cDNA of the SADS-CoV strain (GenBank accession number: MG557844) was divided into eight overlapping fragments and inserted into the pGF vector, a circular yeast artificial chromosome (YAC), via transformation-associated recombination (TAR) (28) (Fig. 2A). Recombinant SADSr-CoV was then constructed by replacing the S gene sequence with that of bat SADSr-CoV (Fig. 2B). YAC plasmids containing the viral genome cDNA and N gene were transfected into Huh7 cells. Five days post-transfection, the supernatant was collected for the blind passaging in Huh7 cells. Significant cytopathic effects (CPEs) were observed in the inoculated Huh7 cells after two passages (P2), and high levels of N protein expression were detected using immunofluorescence assay (IFA) (Fig. 2C), Western blotting (Fig. 2D), and reverse transcription-quantitative PCR (RT-qPCR) (Fig. 2E). These results indicate that the wild-type and recombinant viruses were successfully rescued in Huh7 cells.

### Cell tropism of recombinant SADSr-CoVs

**Fig 3:**
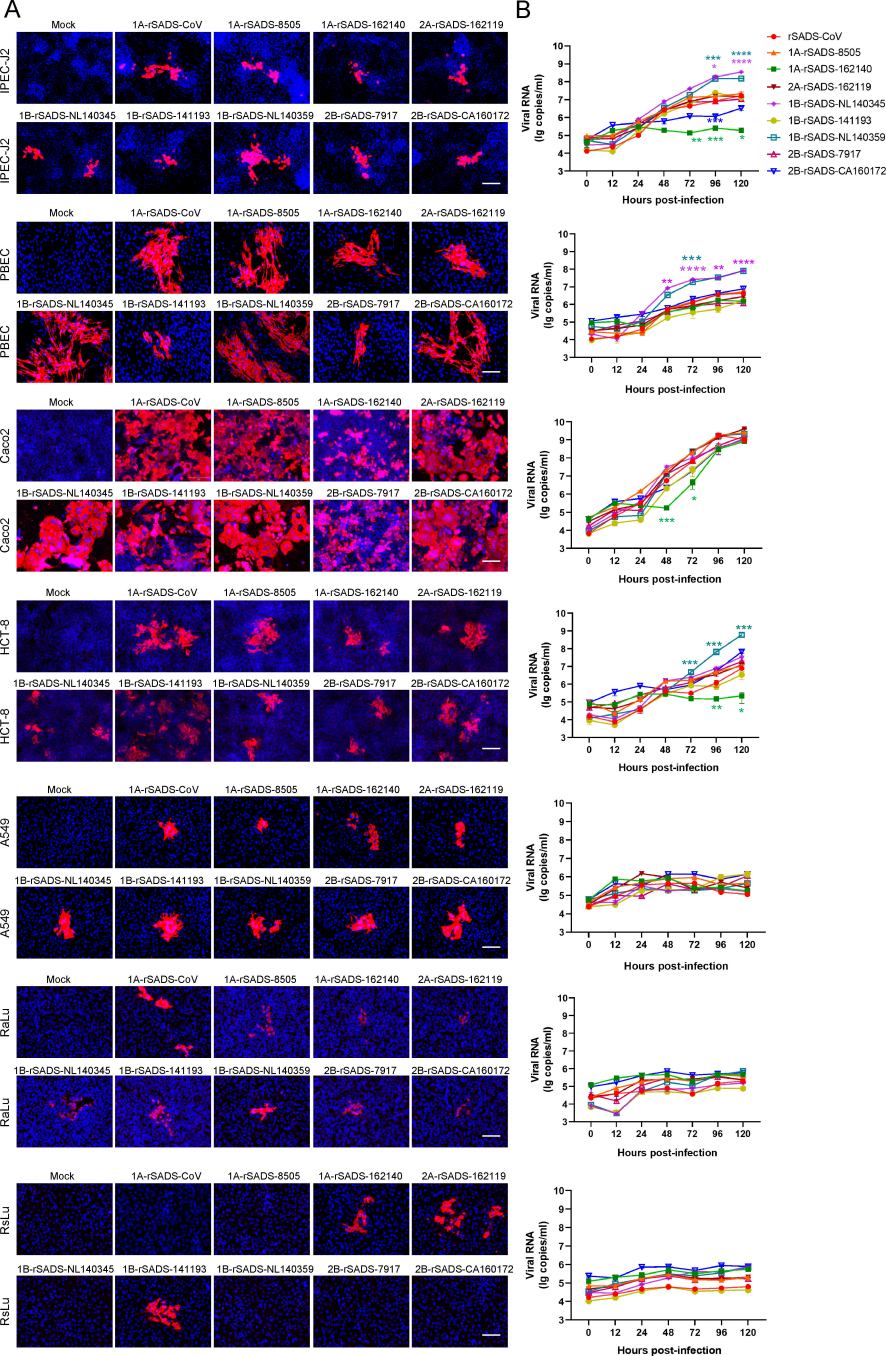
Cell tropisms and replication efficiency of recombinant SADS-CoVs were determined by IFA and RT-qPCR. Intestinal and/or respiratory cell lines derived from swine, human, and bat were inoculated in triplicate with virus at a multiplicity of infection = 0.1 in the absence of trypsin. (**A**) The expression of N protein was assessed by IFA at 72 hpi. Scale bars, 100 µm. (**B**) Supernatant was harvested at 0, 12, 24, 48, 72, 96, and 120 hpi for viral load detection by RT-qPCR assay targeting the viral RdRp gene. Three independent biological replicates were performed with two technical repeats per biological replicate. Values were presented as mean ± SD. An asterisk of specific color denotes a significant difference between the corresponding recombinant SADS-CoV and rSADS-CoV (*P  <  0.05, **P  <  0.01, ***P  <  0.001, ****P < 0.0001).

To determine the host range and transmission potential of bat SADSr-CoVs, we tested seven intestinal and respiratory cell lines derived from different host species, including swine (IPEC-J2 and PBEC cells), humans (Caco-2, HCT-8, and A549 cells), and bats (RaLu and RsLu cells). Cell susceptibility and viral replication efficiency were determined using IFA and RT-qPCR (Fig. 3A and B). All tested swine and human cell lines supported the infection of these viruses; however, there were differences in their replication efficiency. The recombinant viruses carrying the S genes from bat samples 140345 and 140359 (genotype 1B) showed the highest replication efficiency in both swine cell lines. The other viruses, including SADS-CoV, showed similar replication efficiency in PBECs, but varied in IPEC-J2 cells. Similar to SADS-CoV, all recombinant viruses carrying bat SADSr-CoV S genes had a high replication efficiency in Caco2 cells, with a high viral load of 10^10^ copies/mL at 120 h post-infection (hpi) (Fig. 3B); however, they showed low replication efficiency in A549 cells. Because *R. affinis* and *R. sinicus* are the major reservoirs of bat SADSr-CoVs, we also tested viral susceptibility in *R. affinis* and *R. sinicus* lung cell lines (RaLu and RsLu, respectively). RaLu cells were slightly susceptible to SADS-CoV and bat SADSr-CoV infection, resulting in an approximately 1 log_10_ increase in the viral load. RsLu cells were slightly susceptible to 1A-rSADS-162140, 1B-rSADS-NL140345, and 1B-rSADS-NL140359, whereas no infection was detected with the other bat SADSr-CoVs (Fig. 3A and B).

### Replication kinetics of recombinant SADSr-CoV in human and swine organoids

**Fig 4:**
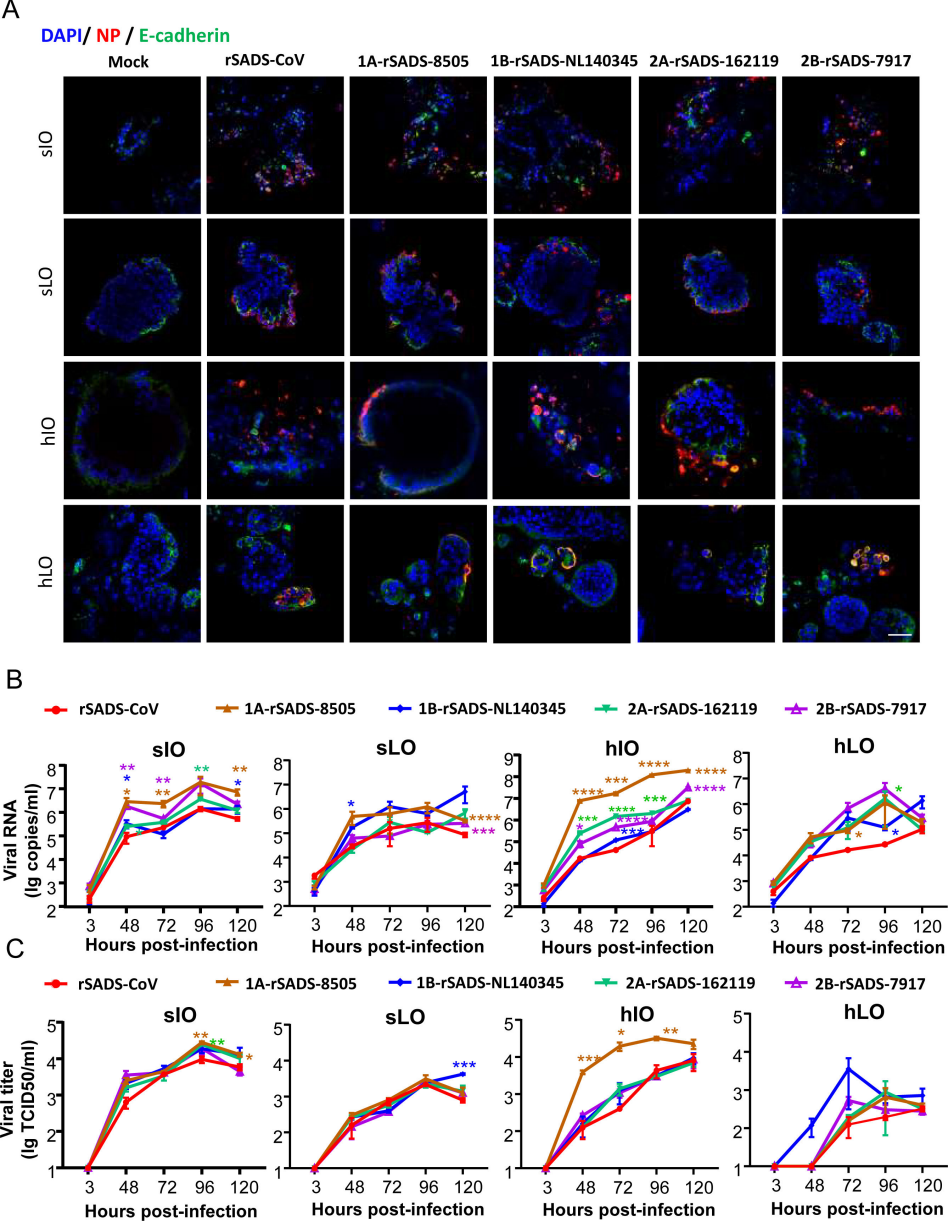
Susceptibility and replication dynamics of recombinant SADS-CoVs in swine and human organoids. (**A**) Intestinal and distal lung organoids derived from human (hIO and hLO) and swine (sIO and sLO) were infected with SADS-CoV and recombinant SADSr-CoVs at an MOI = 3 in the absence of trypsin. Infected organoids were collected and fixed at 72 hpi and immunolabeled with rabbit antibody against the SADS-CoV nucleocapsid protein (red). Epithelial adherens junction and nuclei were stained with E-cadherin (green) and DAPI (blue), respectively. Scale bars, 100 µm. (**B and C**) Intestinal and distal lung organoids were inoculated in triplicate with virus at an MOI = 0.1 in the absence of trypsin. Then, the supernatant was harvested at 3, 48, 72, 96, and 120 hpi. The replication dynamics of chimeric SADSr-CoVs in organoids were determined by RT-qPCR (**B**) and 50% tissue culture infectious dose assays (**C**). Three independent biological replicates were performed in panels B and C. Error bars represent the mean ± SD. An asterisk of specific color denotes a significant difference between the corresponding recombinant SADS-CoV and rSADS-CoV (*P < 0.05, **P < 0.01, ***P < 0.001, ****P < 0.0001).

We further tested the infection and replication efficiency of recombinant SADSr-CoV in lungs and intestinal organoids derived from pigs and humans. Four types of organoids, including swine intestinal organoids (sIO), human intestinal organoids (hIO), swine lung organoids (sLO), and human lung organoids (hLO), were infected with SADS-CoV and four recombinant viruses carrying the S genes representing the four subgenotypes. The infection experiments consisted of two independent sections: IFA staining and viral replication determination. In IFA staining, the organoids were infected with viruses using a multiplicity of infection (MOI) of 3, and the viral N protein expression was detected at 72 hpi. In viral replication assays, the organoids were infected with viruses at an MOI of 0.1, and the extracellular viral RNA levels were measured by RT-qPCR targeting RNA-dependent RNA polymerase gene, and the viruses were titrated in the supernatant from 3 to 120 hpi (Fig. 4A). Substantially increased viral loads were found in the supernatants, with slightly increased loads inside the cells, suggesting that SADS-CoV and the four recombinant bat SADSr-CoVs replicated efficiently in all four organoids (Fig. 4B). We observed more efficient viral replication in the intestinal organoids than in the lung organoids. In addition, 1A-rSADS-8505 showed higher replication efficiency in hIO than SADS-CoV or the other three SADSr-CoVs (Fig. 4C). Taken together, these results collectively demonstrated that four bat recombinant SADSr-CoVs showed productive infection and exhibited replication fitness comparable to that of SADS-CoV in lung and intestinal organoids derived from swine and humans.

### Pathogenicity of recombinant bat SADSr-CoV in suckling mice

**Fig 5:**
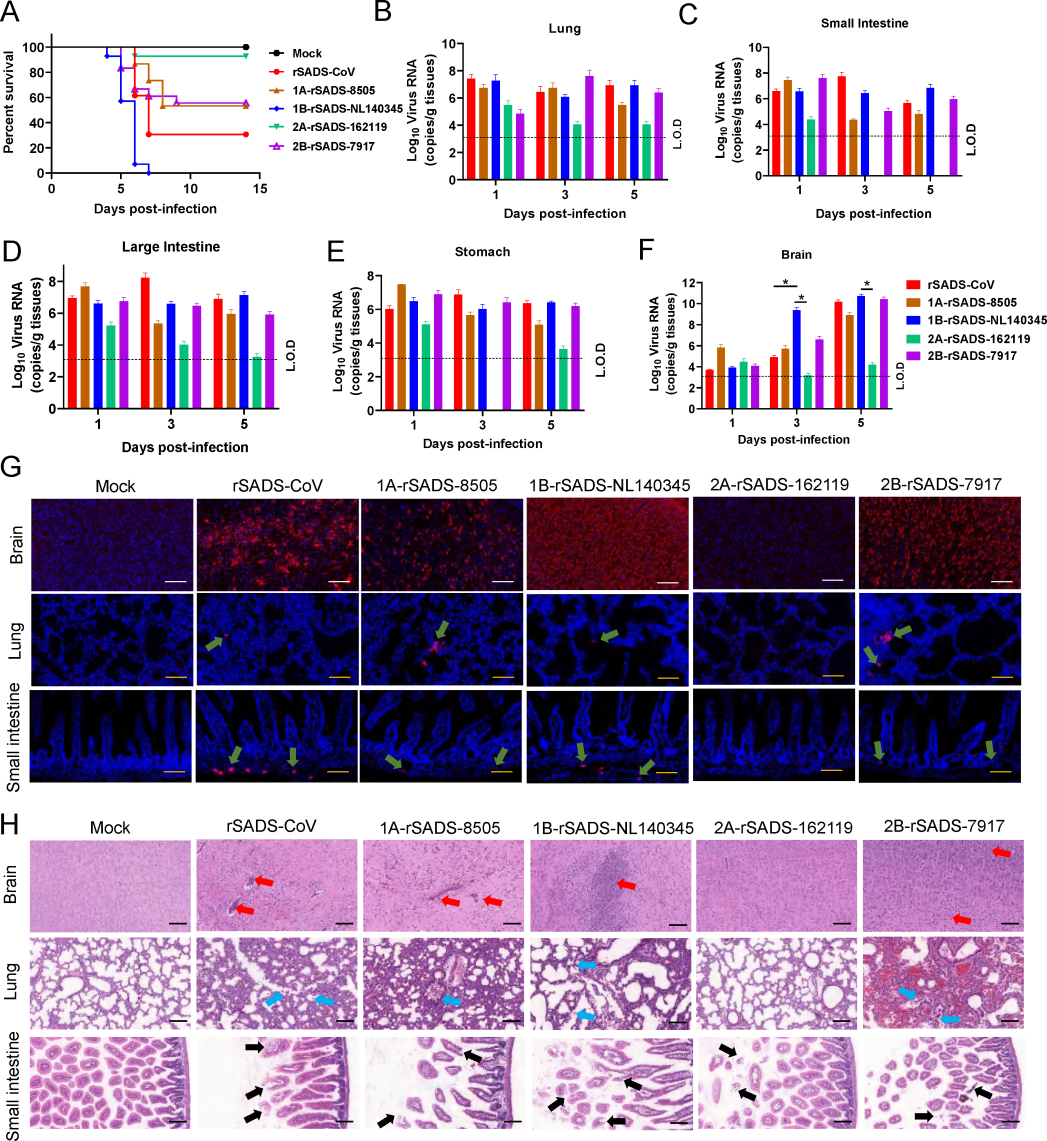
The pathogenicity of recombinant SADSr-CoVs in suckling mice. (**A**) Survival rates of virus-infected mice. The 3-day-old suckling C57BL/6J mice were intragastrically inoculated with 5 × 10^5^ TCID_50_ SADS-CoV (*n* = 13), 1A-rSADS-8505 (*n* = 15), 1B-rSADS-NL140345 (*n* = 14), 2A-rSADS-162119 (*n* = 14), and 2B-rSADS-7917 (*n* = 16) or an equal volume of Dulbecco's modified Eagle medium (*n* = 11) and monitored daily. (**B–H**) The 3-day-old suckling mice were intragastrically inoculated as described in panel **A** and sacrificed for tissue collection at 1, 3, and 5 dpi. Viral RNA in the lung (**B**), small intestine (**C**), large intestine (**D**), stomach (**E**), and brain (**F**) was determined using RT-qPCR. L.O.D, limit of detection. Error bars mean standard error from three technical repeats. Black underline represents the comparison between the indicated groups (*P < 0.05). (**G**) The expression of viral N protein in brain, lung, and small intestine collected at 5 dpi was detected by immunofluorescence. White scale bars, 100 µm. Gold scale bars, 200 µm. (**H**) Hematoxylin and eosin staining was used to observe the pathological changes in the brain, lung, and small intestine collected at 5 dpi. Black scale bars, 100 µm.

Our previous study demonstrated that suckling mice were highly susceptible to SADS-CoV infection and could be used to investigate the pathogenesis of bat SADSr-CoVs (25). Herein, we challenged 3-day-old suckling mice with SADS-CoV and recombinant viruses used for organoid infection (Fig. 5A). The animals were intragastrically infected with 4 × 10^5^ 50% tissue culture infectious dose (TCID_50_) of each virus and observed for 14 days. Infected animals showed a mortality between 7% and 100%. The highest mortality rate (100%) was observed in the 1B-rSADS-NL140345 group. Similar mortality rates were observed for the 1A-rSADS-8505 (47%) and 2B-rSADS-7917 (45%) groups, whereas the 2A-rSADS-162119 group had the lowest mortality (7%). Viral RNA was detected in the lungs, small and large intestines, and stomach of animals infected with SADS-CoV and recombinant viruses rSADS-8505, rSADS-140345, and rSADS-7917 until 5 dpi (Fig. 5B through F). Viral RNA in the brain was detected at a low level at 1 dpi, increased at 3 dpi, and reached a high level at 5 dpi in animals infected with SADS-CoV and the recombinant viruses rSADS-8505, rSADS-140345, and rSADS-7917. However, in the 2A-rSADS-162119-infected group, viral RNA was detectable in multiple tissues at 1 dpi and decreased to the limit of detection or undetectable by 3 or 5 dpi (Fig. 5F). Notably, a 2–4 log higher viral RNA level was detected in the brain than in other tissues, particularly in the brains of the 1B-rSADS-NL140345-infected group at 3 dpi, which was much higher than that in the other four groups, reaching 10^10^ copies/g tissue. Accordingly, a much higher viral N protein expression level was observed in the brains of virus-infected mice at 5 dpi (Fig. 5G). Consistent with previous studies, the level of viral replication in the brain was associated with disease severity, potentially because the virus induces a robust pro-inflammatory immune response in the brain (25, 29).

To further characterize tissue damage in infected mice, we performed histopathological analysis of brain, lung, and small intestine samples at 5 dpi (Fig. 5H). A large amount of neuronal cell necrosis, an increased number of microglia in the brain, neutrophil exudation, and epithelial cell exfoliation were observed in the lungs of mice infected with the three bat SADSr-CoVs (rSADS-8505, rSADS-NL140345, and rSADS-7917) and SADS-CoV; only mild pathological changes were found in 2A-rSADS-162119-infected mice. In addition, atrophy and rupture of intestinal villi were observed in the intestines of all virus-infected mice.

### Detecting cross-neutralization using immune serum

**Fig 6:**
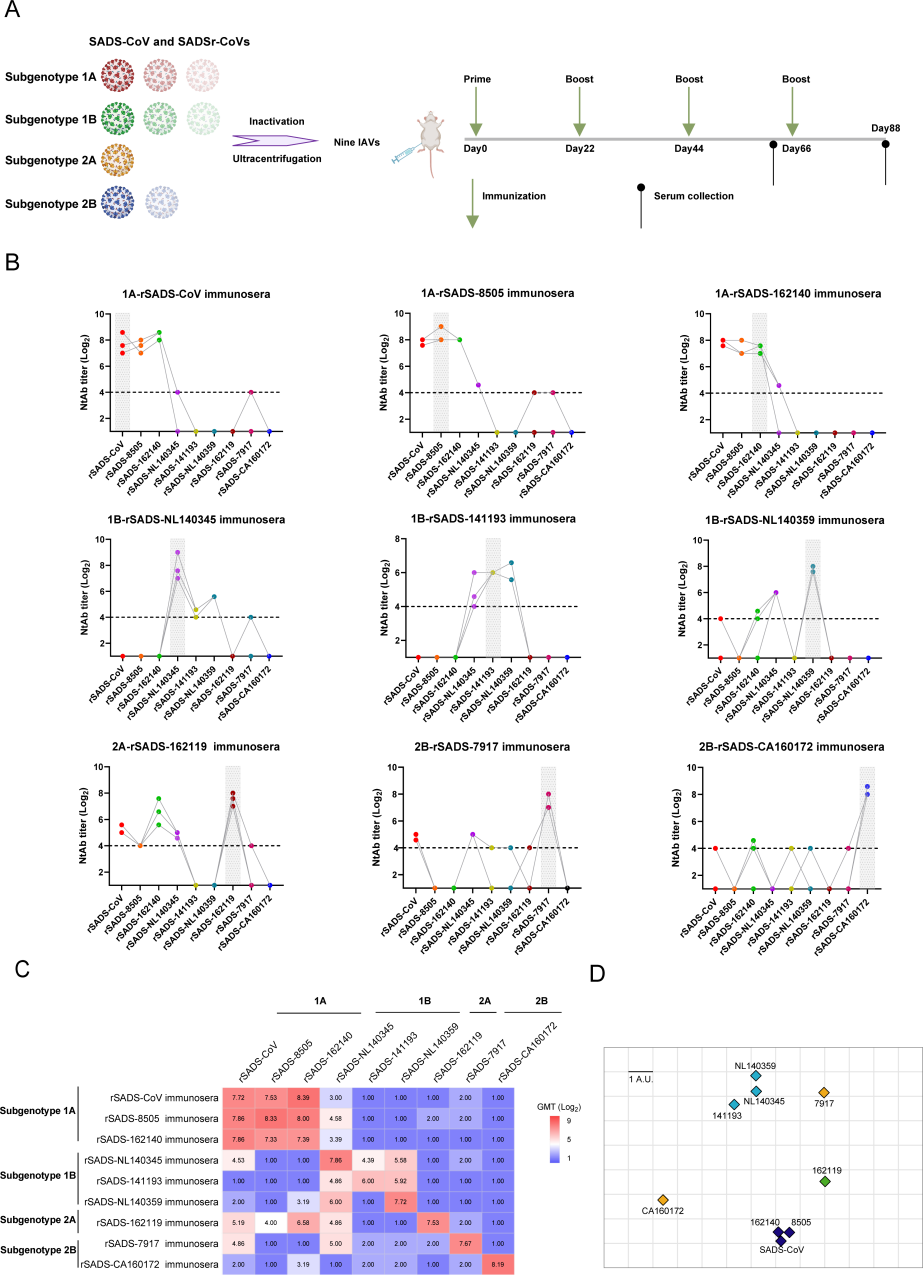
Cross-neutralization assays and antigenic distances among the SADS-CoV and bat SADSr-CoVs. (**A**) Scheme of inactivated vaccine (IAV) preparation and immunization. BALB/c female mice, aged 6–8 weeks, were immunized via intramuscular injection into the thigh muscle. A schematic timeline of the immunization and serum collection schedule in mice is shown. (**B**) Cross-neutralization test of mouse sera immunized with nine inactivated vaccines as described (**A**) in this study. Neutralizing antibody titers were measured in Huh7 by microneutralization test. The result of immune serum against the corresponding virus was highlighted in gray. The detection limits are indicated as dashed lines. (**C**) Heatmap analysis of cross-neutralization activity among SADS-CoV and SADSr-CoVs according to the two-way neutralization titers. Geometric mean titer (GMT) was calculated from the antibody titers of the serum collected from three mice in each group. (**D**) Map of variants in antigenic space. The 2D plots visualize the results of the principal component analysis, mapping serum/virus strain pair neutralization titers into antigenic space. Scale bar indicates 1 A.U., i.e., one arbitrary unit, indicating one unit of antigenic distance and representing twofold change in neutralization titer.

In light of the genetic diversity observed in the spike protein, we assessed the serum cross-neutralization activity among the representative recombinant viruses (Fig. 6A). After four rounds of immunization, each of the nine inactivated viruses induced relatively high titers of neutralizing antibodies (1:64–1:321; Fig. 6B). Two-way cross-neutralization activity was detected within subgenotype 1A viruses at high titers (Fig. 6C). 1B-rSADS-NL140345 and 1B-rSADS-NL140359 immunosera showed limited cross-neutralization activity with each other and low or no neutralization activity against 1B-rSADS-141193. However, 1B-rSADS-141193 immunoserum showed cross-neutralization activity against both 1B-rSADS-NL140359 and 1B-rSADS-NL140345, with a higher titer in 1B-rSADS-NL140359 than in 1B-rSADS-NL140345. No cross-neutralization activity was detected between the two subgenotype 2B viruses. Limited cross-neutralization activity was detected among the four subgenotype viruses, except for low levels of cross-neutralization titers between the 2A-rSADS-162119 immunosera and three 1A viruses, which shared high sequence similarity (97%) in their S1-NTD regions (Fig. 6B and C).

### Antigenic cartography

Using the neutralization data and model (log_2_ titer values), we constructed antigenic maps for the four subgenotypes (Fig. 6D). Subgenotype 1A viruses clustered closely together, similar to the subgenotype 1B viruses. However, SADSr-7917 and SADSr-CA160172 in subgenotype 2B were considerably distant from each other. Ultimately, these SADSr-CoVs were categorized into five serotypes based on their antigenic distances.

## DISCUSSION

Zoonotic pathogens pose significant threats to human health (9, 30–32). Bats are important reservoir hosts of a wide variety of viruses (33–35). To date, more than 16,600 bat-associated viral sequences belonging to at least 31 viral families have been detected in bat populations (36). CoVs account for a high proportion of the 8,255 sequences reported to date (). The spillover risk assessment of known and unknown viruses from wildlife is important for active surveillance of domestic animals and humans and for preparing countermeasures in advance.

In this study, we analyzed 69 novel bat SADSr-CoV S genes that showed high genetic diversity in the S1 region but were highly conserved in the S2 region (Fig. S1). Together with previously reported S sequences from bat SADSr-CoV and SADS-CoV, we classified them into four subgenotypes based on variations in their S1-NTD and S1-CTD sequences. Bat viruses with S genes closely related to SADS-CoV were identified not only in Guangdong Province in China, where the SADS outbreak was first reported, but also in Yunnan Province. To compare the functions of the different S proteins, we constructed an infectious cDNA clone of SADS-CoV and a series of recombinant SADS-CoVs by replacing the S genes with those of bat SADSr-CoV. We demonstrated that all bat-derived recombinant viruses showed high replication efficiency similar to SADS-CoV in Caco2 cells and could replicate in other tested cell lines from swine and humans. In particular, cell lines derived from the intestines were found to be more susceptible than those from the lungs. Among them, two subgenotype 1B viruses (NL140345 and NL140359) exhibited higher replication efficiencies than other SADSr-CoVs in IPEC-J2 and PBEC cells (porcine intestinal and bronchial epithelial cell lines, respectively); however, this difference was not observed in our tested organoid models (Fig. 3A, B, 4B and C). A previous study showed that porcine intestinal organoids derived from the duodenum, jejunum, and ileum effectively supported the growth of SADS-CoV (37). In our study, the replication kinetics of SADSr-CoVs were tested in intestinal and lung organoids derived from swine and humans (Fig. 4B and C). We demonstrated that the tested viruses replicated more efficiently in intestinal organoids than in lung organoids, consistent with the higher viral titers detected in intestinal cell lines (Caco2 and HCT-8 cells). Our results indicate that these bat SADSr-CoVs can potentially infect both the respiratory and intestinal tracts of pigs and humans but exhibit a more pronounced preference for intestinal infections.

In a previous study, we have demonstrated that SADS-CoV is lethal to suckling mice. Although the suckling mouse model did not perfectly replicate the tissue tropism observed in SADS-CoV-infected piglets, it provides a preliminary cost-effective model to investigate the pathogenesis of SADS- and SADSr-CoVs (25). Thus, in this study, we evaluated the pathogenicity of recombinant viruses carrying different bat SADSr-CoV S genes in suckling mice. Our results revealed that these viruses exhibited different mortality rates, with genotype 1 strains exhibiting higher overall mortality than genotype 2 strains. The brain is the primary target organ of SADS-CoV and SADSr-CoVs in suckling mice; the intestines showed only mild pathological changes after infection. In this study, we observed a distinct phenotype between *in vitro* and *in vivo* studies for 2A-rSADS-162119: similar cell tropism and replication ability to other recombinant viruses *in vitro* (Fig. 3 and 4) but different pathogenicities *in vivo* (Fig. 5 and 6). Our results demonstrate the importance of using live viruses in animal models to assess the pathogenesis of novel viruses identified in wildlife. Further studies should be performed to understand the mechanism underlying viral pathogenesis contributed by different bat SADSr-CoV S proteins, which will be helpful for the development of attenuated vaccines.

The CoV S protein plays a significant role in receptor binding during entry into host cells and induces the neutralization of antibodies in the host (38). Previous studies have shown that the receptor binding domain of SADS-CoV may be located in the S1-CTD region (39, 40). In the present study, we performed a two-way serum cross-neutralization assay using SADS-CoV and bat SADSr-CoV hyperimmune sera. Our results show that recombinant SADS-CoVs, which share high sequence similarity in their S1-CTD regions, had high cross-neutralization activity. We also observed low neutralization activity of subgenotype 1A viruses to 1B virus serum, even though these two subgenotypes share high similarity (>97%) in the S1-NTD region (Table S1). These results further demonstrate that epitope-inducing neutralizing antibodies may primarily reside in S1-CTD. Our two-way cross-neutralization assay results showed that the known bat SADSr-CoVs and SADS-CoV could be classified into five neutralization serotypes. Considering the pathogenicity and potential spillover risk of these viruses, it is necessary to develop broad-spectrum antiviral drugs and design a vaccine that induces a broad cross-protection against this group of viruses.

Although the direct transmission of bat coronaviruses to humans rarely causes epidemic, indirect transmission to humans from bats through intermediate animals is believed to cause severe epidemics and global pandemics, such as SARS, MERS, and SARS-CoV-2 (4, 7, 8, 41). Pigs, which are in close contact with humans and other animals, have been recognized as intermediate hosts for various viruses, including avian influenza A and the Nipah virus (42, 43). Moreover, several porcine deltacoronavirus strains have recently been identified in plasma samples from three Haitian children with acute undifferentiated febrile illness (44), further addressing concerns about swine-to-human transmission of coronaviruses. Our findings show that SADSr-CoVs circulating in *Rhinolophus* bats pose a potential risk for interspecies infection. Therefore, it is imperative to continue the surveillance of this group of viruses in swine and other animals.

Our results revealed that different spike genes induce different pathogenicity in suckling mice, emphasizing the importance of closely monitoring potentially virulent strains. However, the pathogenicity of SADSr-CoV should be further evaluated in piglets in future studies. Importantly, while the S gene of CoV plays a critical role in cell tropism and virulence, overall virulence is likely mediated by multiple viral genes. To comprehensively evaluate the pathogenicity of bat SADSr-CoVs, future studies should assess the entire viral genome, rather than focusing solely on the S gene. Additionally, our previous study showed that immunized female mice provided partial protection to their offspring from homologous challenge. Further investigation into heterologous protection *in vivo* is needed to confirm our cross-neutralization findings.

## MATERIALS AND METHODS

### Sample collection and viral detection

Bat fecal swab or fresh fecal samples were collected in viral transport medium as described previously (2, 6). Bat species were identified by targeting the *Cytb* gene. Detailed information, including bat species, sampling location, collection year, and sample ID, was provided in Fig. S1. Viral RNA was extracted from bat anal samples and analyzed for the presence of bat SADSr-CoV using family-specific degenerate semi-nested PCR targeting the partial RdRp sequence (15).

### Amplification and sequencing of the spike gene

Primers targeting the spike gene were designed based on the alignment of reported SADS-CoV and related coronavirus sequences (primer sequences provided upon request). Nest-PCR amplification was performed as described previously (45). The expected PCR products were gel-purified and sequenced directly using the target primers. Weak bands were cloned into the pGEM T-easy vector and sequenced using Sanger ABI-PRISM.

### Construction of viral genome cDNA clones using TAR

The SADS-CoV genome was divided into eight overlapping cDNA fragments, each with 40–100 bp overlaps. Viral cDNA fragments were obtained via RT-PCR amplification of RNA extracted from the viral isolates using a SuperScript IV One-Step RT-PCR System. *Bsa*I sites were introduced at both the 5′ and 3′ ends of the viral cDNA during PCR. Additionally, the cytomegalovirus (CMV) promoter and HDV ribozyme amplified from pBAC-CMV (46) were introduced into the 5′-UTR and 3′-UTR ends of the viral cDNA, respectively. In addition to the B and C1 fragments, other PCR products were purified and cloned into the pGEM-T-EASY vector. Subclones or fragments (A–G) were digested with *Bsa*I, then all digestion products were separated on 1% agarose gels, excised, and purified using a gel extraction kit (Omega, USA). The vector pGF was used for TAR cloning, which was amplified via PCR using primers containing 40 bp overlaps of fragments encompassing the 5′- or 3′-ends of the SADS-CoV genome (28). DNA mixtures containing viral cDNA and vector fragments were prepared for yeast transformation as described in previous studies (47, 48). At least five yeast clones were randomly selected and grown on an SD-His liquid medium. Positive colonies were screened using PCR targeting multiple fragment junctions.

### Isolation and purification of circular YACs containing the viral genome

Circular YACs containing the viral genome were isolated as described previously (49). At least three yeast clones were grown in 1 L of fresh medium. The yeast cells, harboring circular YAC, were collected and digested using Zymolyase 20T with β-mercaptoethanol, followed by treatment with a lysis buffer (0.05 M Tris-HCl, 0.02 M EDTA, and 1% SDS [pH 12.8]). The YACs were then isolated via chloroform extraction and isopropanol precipitation. Circular YAC DNA was purified using a large-construct kit (Qiagen, Germany) according to the manufacturer’s instructions. Supercoiled YAC DNA was analyzed via 1% (wt/vol) agarose gel electrophoresis. Each isolated YAC clone was sequenced using next-generation sequencing.

### Rescue of infectious clones

Given that Huh7 cells exhibited a good transfection rate and were susceptible to SADS-CoV infection, infectious YAC clones were transfected into Huh7 cells using Lipofectamine 3000 (Invitrogen, USA) according to the manufacturer’s protocol. Huh7 cells were seeded in six-well plates 1 day in advance. The next day, when cells were 70%–90% confluent, 6 µg of infectious YAC plasmids or other modified YACs were diluted in Opti-MEM with P3000 reagent (Thermo Fisher, USA) and mixed with Lipofectamine 3000 reagent (Invitrogen). The DNA transfection solution was mixed well by gently tapping the tube, incubated for 15 min at room temperature to allow the formation of DNA-lipid complexes, and then added dropwise onto the cells. Transfected cells were incubated at 34°C in BSL-2 conditions. At 6 h post-transfection, the supernatant was removed and replaced with fresh serum-free Dulbecco's modified Eagle medium (DMEM) containing 0.5 µg/mL trypsin. At 5 days post-transfection (dpt), the supernatant was collected for the serial passaging of Huh7 cells. The CPEs were monitored daily.

### Confirmation of recombinant SADSr-CoVs

Three analyses were performed to confirm the rescue of the recombinant SADSr-CoV from the YAC infectious clone system. First, Huh7 cells were collected at 5 dpt; the total RNA was extracted using a TRIzol kit; and the production of intracellular viral subgenomes was confirmed by RT-PCR (50). Second, after two passages (P2), significant CPEs were monitored and recorded. The cells were then fixed at 5 dpi, and the SADS-CoV N protein was detected using an appropriate antibody via IFA (51). Third, productive infection with SADS-CoV and rSADS-CoV was detected in Huh7 cells. Briefly, Huh7 cells were incubated with 100 µL supernatant from the rescued viruses at P2, and the supernatant was harvested at 0, 2, and 4 dpi for viral RNA extraction and RT-qPCR, as described previously (52). The expression of the N protein was detected using Western blotting in Huh7 cells infected with the third-passage virus (P3) at 2 dpi.

### Genetic characterization of stock viruses

The genetic identity and integrity of the stock viruses used for challenge experiments were confirmed by sequencing the complete S glycoprotein gene. Viral RNA was extracted from supernatants of inoculated Huh7 cells and served as the template for nested-PCR amplification of the S gene using the methods described above. The amplified products were purified and subjected to Sanger sequencing. Finally, the resulting consensus sequences were aligned to their corresponding reference sequences to verify their identity and genetic stability. The full list of mutations is provided in Table S2.

### Establishment and maintenance of intestinal and airway organoids from humans and swine

Intestinal and airway organoids from humans and swine were used to assess susceptibility to SADS-CoV. Establishment, maintenance, and differentiation of human intestinal and airway organoids were performed based on our previous reports (53, 54). Porcine ileum organoids were prepared according to the protocol reported by Zhang et al., with modifications (55). Briefly, intestinal sections were cut longitudinally and opened, and the mucosal layer and villi were removed by scraping using a microscope slide. Subsequently, 2–4 cm segments of the intestinal sections were cut into 5 mm-long pieces. The dissected pieces underwent 5–10 washes with phosphate-buffered saline (PBS) until the supernatant became clear. After washing, the pieces were incubated in cold PBS supplemented with 2.5 mM EDTA for 40 min at 4°C with gentle rotation. The pieces were then pelleted and vigorously pipetted up and down in 10 mL of cold PBS to release the crypts. The supernatant containing crypts was centrifuged at 400 × *g* for 3 min. The crypts were washed twice with cold PBS and suspended in Matrigel. This crypt-Matrigel suspension was then dispensed in 50 µL aliquots into the center of each well of a 24-well plate. The spent culture medium was changed every 4 days, and the organoids were passaged every 2 weeks.

Porcine airway organoids were obtained from two piglet donors using previously described protocols (56), with minor modifications. Briefly, fresh lung tissues were washed with 20 mL prechilled PBS several times in a 50 mL centrifuge tube until the supernatant became clear. Then, the lung tissue was carefully cut into 2 mm-long segments and incubated at 37°C for 1–2 h in a mixture of 400 U/mL collagenase (Gibco, USA) and airway organoid medium at 37°C for 12 h. Following incubation, the suspension underwent centrifugation at 250 × *g* at 4°C for 5 min. The resulting pellet of lung cells was resuspended in ice-cold PBS containing 5% fetal bovine serum and then filtered through a 100 µm filter to obtain airway cell pellets. The pelleted lung cells were then resuspended in cold Matrigel and seeded in 24-well plates. After the Matrigel solidified, 500 µL of airway organoid medium was added to each well, and the plates were transferred to a humidified 5% CO_2_ incubator at 37°C. The spent culture medium was changed every 4 days, and the organoids were passaged every 2 weeks.

### Organoid susceptibility assays

After culturing for 2 weeks, 3D airway organoids were washed gently with ice-cold 0.5% EDTA-PBS and harvested in a 50 mL centrifuge tube via centrifugation at 400 × *g* for 5 min at 4°C. The harvested organoids were incubated with a cell recovery solution to remove the Matrigel and then digested with TrypLE to break them into large pieces. An appropriate amount of organoids was taken and digested into single cells for cell counting. The remaining sample can be incubated on ice to maintain cell viability. Then, SADS-CoV and recombinant SADSr-CoV infections were performed at an MOI of 0.1 for viral replication determination and at an MOI of 3 for IFA staining. After a 2 h incubation for virus adsorption at 34°C, the organoids were washed twice with PBS to remove the unbound virus. The pellet was then resuspended in cold Matrigel and placed as 45 µL droplets into each well of a 24-well plate. After the Matrigel solidified, 500 µL culture medium was added to each well. At the indicated time points, the supernatants and cell lysates were harvested for viral RNA extraction, RT-qPCR, and TCID_50_ assays. All TCID_50_ assays were performed in Huh7 cells and calculated using the Reed–Muench method.

### Immunofluorescent staining of organoids

Immunofluorescence staining of organoids was performed as previously described (53). Briefly, all organoids were gently scraped, detached from the wells, and then fixed with 4% paraformaldehyde at 4°C for 24 h. After fixation, the organoids were washed with PBS, permeabilized with 0.1% Triton X-100, and incubated with rabbit anti-SADS-CoV N polyclonal antibodies and mouse anti-E-cadherin (BD Biosciences, USA), followed by incubation with Cy3-conjugated antirabbit IgG and FITC-conjugated anti-mouse IgG. Finally, the organoids were imaged using a confocal microscope (Leica STELLARIS 8, Germany).

### Experimental infection of suckling mouse with recombinant SADSr-CoVs

Mouse infection experiments were performed via intragastric administration, as previously described (25). Pregnant C57BL/6J mice were purchased from Hunan SJA Laboratory Animal Co., Ltd., and housed in an ABSL2 facility, under specific pathogen-free conditions. Following gestation, 3-day-old C57BL/6J mice were inoculated via the intragastric routes with 4 × 10^5^ TCID_50_ of SADS-CoV, recombinant SADSr-CoVs, or an equal volume of DMEM. For the survival monitoring experiment, mice were observed daily for clinical signs over a 14 day period. For the pathology progression experiment, three or four C57BL/6J mice were euthanized at 1, 3, and 5 dpi, and tissues, including the lung, small intestine, large intestine, stomach, and brain, were harvested.

### Preparation of inactivated virus

Each virus strain was amplified using 10 T175 cell culture flasks, with cytopathic effects monitored daily. When the cytopathic effect reached approximately 30%, we collected the virus-containing supernatant and centrifuged it at 3,000 × *g* for 20 min at 4°C. β-Propiolactone was then added at a 1:4,000 ratio, gently mixed, and incubated at 4°C for 48 h to ensure maximal inactivation of the virus. Inactivated virus supernatant was concentrated by ultracentrifugation through a 5 mL of 25% Opti-Prep cushion at 27,000 rpm for 2 h using a SW32 rotor (Beckman). After ultracentrifugation, the pelleted viral particles were resuspended in 1 mL PBS and aliquoted 250 µL per tube for use or storage at −80°C.

### Immunization and serum collection of mouse

Diluted aluminum adjuvant (Thermos, USA) was added dropwise to the inactivated virus and gently mixed by pipetting. To ensure effective adsorption of the antigen, mixing was continued on a rotator for 30 min. BALB/c mice aged 6–8 weeks were immunized with 150 µL inactivated vaccine (75 µL of inactivated virus or 75 µL PBS, along with 75 µL of aluminum hydroxide, via intramuscular injection into the thigh muscle, and each inactivated virus strain immunized three mice. Immunization was performed four times in total, with each mouse receiving one dose every 22 days. Microblood was collected before the fourth immunization and used to detect viral antibody production. After 22 days from the fourth immunization, whole blood was collected, and serum was obtained for subsequent neutralization assays.

### Viral neutralization assay

A virus neutralization assay was performed using 96-well plates. Briefly, immunized serum samples from the nine groups of viruses were heat-inactivated at 56°C for 30 min. The immunized sera were then serially diluted at 1:8, 1:16, 1:32, 1:64, 1:128, and 1:256. An equal volume of virus stock (containing 100 PFU) was added to the diluted sera and incubated at 37°C in a 5% CO_2_ incubator. After 1 h of incubation, 100  µL of each mixture was inoculated onto Huh7 cell monolayers for 1 h with shaking. Finally, the supernatant was removed and replaced with fresh serum-free DMEM containing 0.5 µg/mL trypsin. Five days later, the neutralizing antibody titer was calculated using the Reed–Muench method and expressed as the reciprocal of the highest serum dilution at which the CPE in 50% of the wells was completely inhibited.

### Antigenic space plots

The correlation between serum and virus pairs in this experiment was analyzed using principal component analysis (PCA), as described in previous study (57). Briefly, the antigenic distance was calculated based on the neutralization titer data. Nine groups (three mice in each group) of serum were collected and compared against nine virus strains, assembling a 9 × 9 matrix. The values in the serum/virus strain matrix were analyzed via PCA, which decomposed the data to two dimensions. The antigenic spatial distances were then visualized using Racmacs.

### Statistical analysis

Data are presented as mean ± SD and were analyzed using Tukey’s multiple-comparison test in the GraphPad software. Differences with a *P* value of <0.05 were considered statistically significant.

## Data Availability

The data reported in this paper have been deposited in the GenBase in National Genomics Data Center, Beijing Institute of Genomics, Chinese Academy of Sciences/China National Center for Bioinformation, under accession numbers C_AA130023.1 to C_AA130091.1, which are publicly accessible at https://ngdc.cncb.ac.cn/genbase. The data sets generated and/or analyzed during the current study are available from the corresponding author upon request. Source data are provided with this paper.

## SUPPLEMENTAL MATERIAL

Supplemental figures jvi.02240-24-s0001.docx Figures S1 and S2.

Supplemental legends jvi.02240-24-s0002.docx Legends for Tables S1 and S2.

Table S1 jvi.02240-24-s0003.xlsx Amino acid identity of multiple regions, including S1-NTD, S1-CTD, S1, S2, and S, among representative bat SADSr-CoVs.

Table S2 jvi.02240-24-s0004.docx Nonsynonymous mutations in the spike protein of stock viruses.

ASM does not own the copyrights to Supplemental Material that may be linked to, or accessed through, an article. The authors have granted ASM a non-exclusive, world-wide license to publish the Supplemental Material files. Please contact the corresponding author directly for reuse.
