# Supplementary material for: Swine acute diarrhea syndrome coronavirus-related viruses from bats show potential interspecies infection
Source: J Virol. 2025 Nov 19;99(12):e02240-24. doi: 10.1128/jvi.02240-24 (PMC12724316; doi:10.1128/jvi.02240-24)

**Fig.S1** **Amino acid identity of multiple regions, including S1-NTD, S1-CTD, S1, S2 and S, of representative bat SADSr-CoVs were compared with those of SADS-CoV (MG557844) and bat HKU2 prototype strain (NC_009988)**. Amino acid identity was calculated with Megalign using the Clustal W method. The sequences obtained in previous study are labeled in gray.


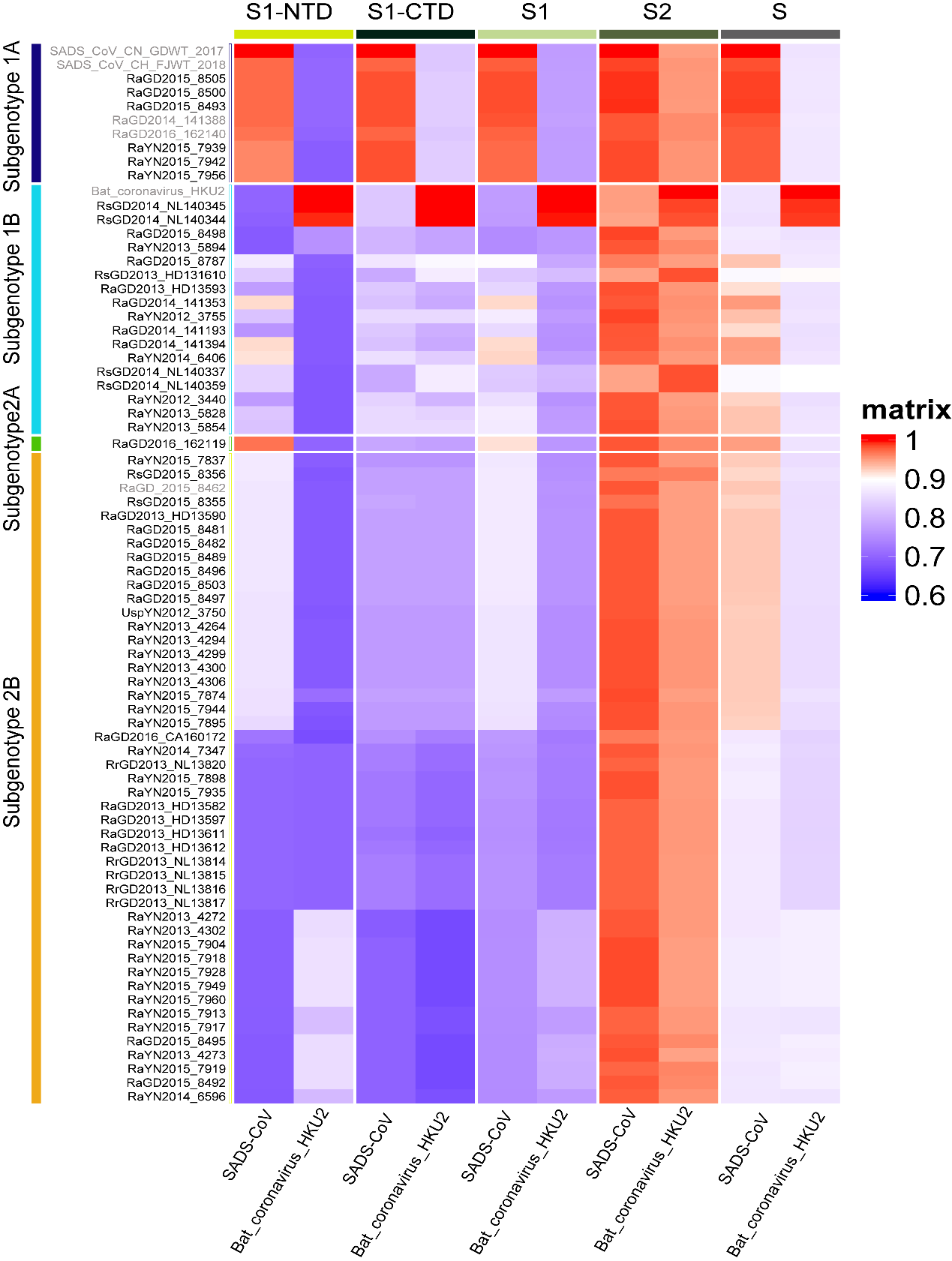


**Fig.S2 Phylogenetic analysis of the SADSr-CoV S1-NTD and S1-CTD in this study**. Multiple amino acid sequence alignments were performed using MAFFT (v.7.508) with default parameters. Phylogenetic trees of S1-NTD (A) and S1-CTD (B) were constructed using the neighbor-joining method in IQ-TREE (v.2.2.0.3) and bootstrap values calculated from 1000 replicates, and visualized in FigTree (v.1.4.3). The sequences of representative strain in this study were labeled in corresponding color.


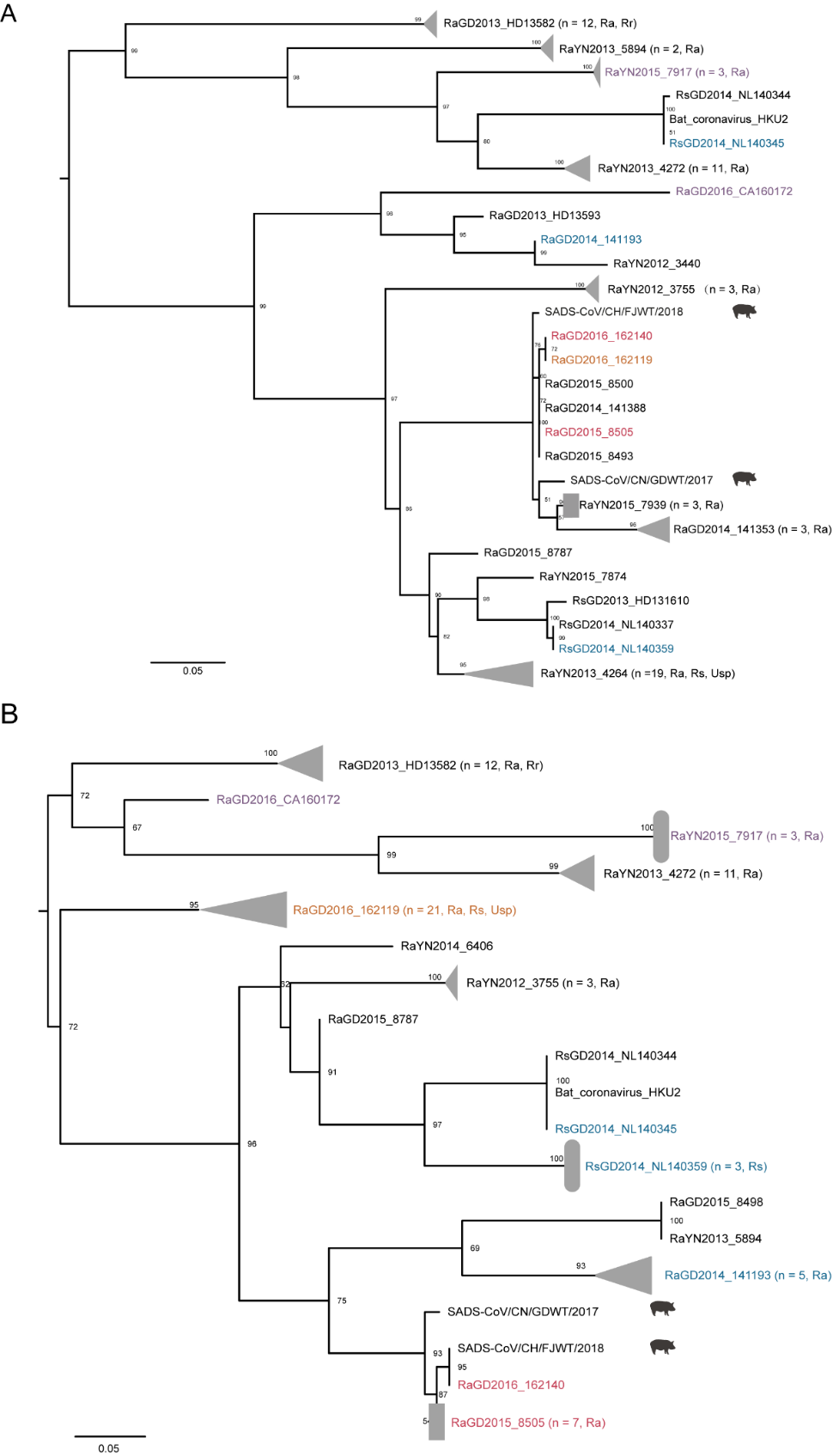

Supplement: Supplemental figures — Figures S1 and S2. [file jvi.02240-24-s0001.docx]
