# Supplementary material for: Swine acute diarrhea syndrome coronavirus-related viruses from bats show potential interspecies infection
Source: J Virol. 2025 Nov 19;99(12):e02240-24. doi: 10.1128/jvi.02240-24 (PMC12724316; doi:10.1128/jvi.02240-24)
Supplement: Supplemental legends — Legends for Tables S1 and S2. [file jvi.02240-24-s0002.docx]

**Supplementary Table 1**

Amino acid identity of multiple regions, including S1-NTD, S1-CTD, S1, S2 and S, among representative bat SADSr-CoVs. The identities of all sequences from different genotypes were calculated with Megalign using the Jotun Hein method.

**Supplementary Table 2**

Nonsynonymous mutations in the spike protein of stock viruses.
