# Supplementary material for: Swine acute diarrhea syndrome coronavirus-related viruses from bats show potential interspecies infection
Source: J Virol. 2025 Nov 19;99(12):e02240-24. doi: 10.1128/jvi.02240-24 (PMC12724316; doi:10.1128/jvi.02240-24)
Supplement: Table S2 — Nonsynonymous mutations in the spike protein of stock viruses. [file jvi.02240-24-s0004.docx]

| Virus strain | Nonsynonymous mutation in the spike gene |
| --- | --- |
| rSADS-CoV | None |
| 1A-rSADS-8505 | Glu 262 Lys |
| 1A-rSADS-162140 | Ala 737 Thr Value 755 Gly |
| 1B-rSADS-NL140345 | Val 831 Asp |
| 1B-rSADS-141193 | Pro 659 Ser |
| 1B-rSADS-NL140359 | None |
| 2A-rSADS-162119 | Asn 892 Tyr |
| 2B-rSADS-7917 | Phe 556 Leu |
| 2B-rSADS- CA160172 | Ser 595 Asn Val 760 Gly |
